# Supplementary material for: Safety and immunogenicity of booster vaccination and fractional dosing with Ad26.COV2.S or BNT162b2 in Ad26.COV2.S-vaccinated participants
Source: medRxiv. 2023 Nov 20:2023.11.20.23298785. Preprint. [Version 1] doi: 10.1101/2023.11.20.23298785 (PMC10690356; doi:10.1101/2023.11.20.23298785)
Supplement: Supplement 1 [file NIHPP2023.11.20.23298785v1-supplement-1.pdf]

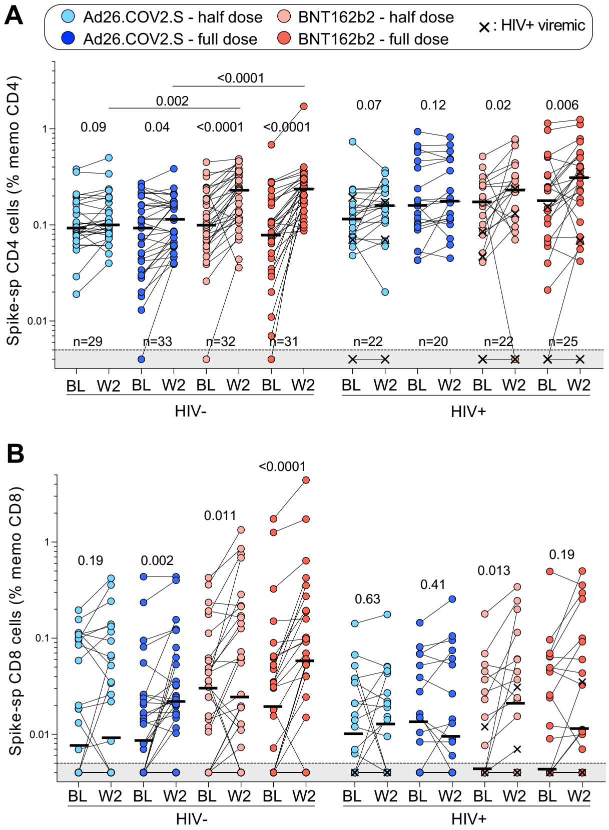

Supplemental Figure 1: SARS-CoV-2 spike-specific T cell responses in participants stratified by HIV status in each study arm. Frequency of spike-specific CD4+ T cells **(A)** and spike-specific CD8+ T cells **(B)** before (BL) and 2 weeks after vaccine boost (W2) in HIV-negative participants and in PLWH. Bars represent medians. Viremic PLWH are identified with a cross. Bars represent medians. A two-tailed Wilcoxon signed-rank test was used to assess statistical differences between paired samples and a Kruskal-Wallis with Dunn's corrections was used to compare different groups.

Supp Table 1

	Vax-naive	J&J primed	J&J boosted
N	14	14	6
Age*	46 (37-57)	46 (37-57)	55 (49-57)
Gender (% female)	100%	100%	100%
Time since last vaccination (days)*	na	44 (31-53)	110 (96-117)

Supplementary Table 1: Clinical characteristics of samples used for Ad26-specific antibody response assessment. (Related to Figure 10F). *: Median and Interquartile range (IQR), na: not applicable.

Supp Table 2

	Vax-naive	J&J primed
N	20	11
Age*	43 (33-53)	49 (32-53)
Gender (% female)	90%	81.8%
Time since vaccination (days)*	na	25 (20-45)

Supplementary Table 2: Clinical characteristics of samples used for Ad26-specific T cell response assessment. (Related to Figures 11D and 11G). All participants were HIV-uninfected. *: Median and Interquartile range (IQR). na: not applicable.
